# Supplementary material for: Biomimetic Silica Particles with Self-Loading BMP-2 Knuckle Epitope Peptide and Its Delivery for Bone Regeneration
Source: Pharmaceutics. 2023 Mar 25;15(4):1061. doi: 10.3390/pharmaceutics15041061 (PMC10142205; doi:10.3390/pharmaceutics15041061)
Supplement: Supplementary file 1 [file pharmaceutics-15-01061-s001.zip › pharmaceutics-2207864-supplementary.pdf]

Supplementary data

Biomimetic silica particles with self-loading BMP-2 knuckle epitope peptide and its delivery for bone regeneration

Mi-Ran Ki[1,2], Thi Khoa My Nguyen[1], Tae-In Park[1], Hae-Min Park[3,*,†], Seung Pil Pack[1,*,†]

[1]Department of Biotechnology and Bioinformatics, Korea University, Sejong 30019, Republic of Korea

[2]Institute of Industrial Technology, Korea University, Sejong 30019, Republic of Korea

[3]Department of Chemical Engineering and Applied Chemistry, Chungnam National University, Daejeon 34134, Republic of Korea

[†]These authors contributed equally to this work.

*Corresponding author:

Seung Pil Pack, Email: spack@korea.ac.kr, TEL: +82-44-860-1419, FAX: +82-44-860-1598

Hae-Min Park, E-mail: haemin.park@cnu.ac.kr, TEL:+82-42-821-5683

Materials and Methods

1. Alkaline phosphatase (ALP) activity

The 100 μL of MC3T3 E1 osteoblastic cells in GM (1 × 10_5 cells mL-1) were seeded onto a well of 96-well plates and then cultured for 24h under GM medium, the medium was replaced with BM in the absence or presence of each indicated P4 preparation. The BM was replaced every 3 days and ALP activity was measured at 7 and 14 days thereafter. rhBMP2 was used for positive control. pNPP solution (Sigma-Aldrich) was used as substrate for ALP activity. The cells were washed with PBS and lyzed with 50μL of PBS containing 1 % Triton X-100 at 37°C for 5min. The lyzed cells were incubated at 37°C for 10 to 30 min depending on color development in the presence of 50 μL of pNPP solution. The absorbance was recorded at 405 nm using a UV/visible microplate reader (Infinite M200 PRO NanoQuant; TECAN).

2. Determination of the peptide binding amount on HA and silica deposition by bound peptide on HA

To prepare peptide-bound hydroxyapatite (HA), 100 mg of HA powder with particle size less than 200 nm was immersed in 1 mL of a solution containing either P4 or P4E×6 peptide (1 mg/mL) and left overnight at 4°C. The unbound peptides were removed by gentle rinsing, and the amount of bound peptide was determined using the Pierce TM Quantitative Peptide Assay kit (Thermo Fisher Co.) by subtracting the amount of unbound peptide from the total added peptide amount. The peptide-bound HA was then reacted in 1 × PBS containing 20 mM hydrolyzed TMOS overnight with gentle agitation. After washing once with ddH2O, the peptide@Si/HA was dried. The amount of silica precipitated by the peptide was determined using a modified molybdenum blue method. The silica precipitate was dissolved in 1 M NaOH at 95°C for 10 min and neutralized with 1 M HCl. Then, a 1% ammonium haptamolybdate solution in 0.1 N sulfuric acid was added to produce a β-silicomolybdate complex (yellow color), followed by the addition of 1% oxalic acid to avoid phosphate interference. Finally, 100

mM ascorbic acid was added to amplify the color development, and the absorbance of the final mixture was measured at 810 nm using a microplate reader (Infinite M200 PRO; TECAN, Austria). Sodium metasilicate was used for standard silica solution.

3. Immunoblot analysis after treatment with P4 peptide in MC3T3 E1 cells

MC3T3 E1 osteoblastic cells in GM (1×10^5 cells mL^{-1}) were seeded onto a well of 6-well plates and then cultured for 24 h under the same conditions as mentioned above, the medium was replaced with BM in the absence or presence of each indicated HA composite. After each treatment, cells were harvested at 4 and 8 hours using 50 μL RIPA buffer (RIPA Lysis Buffer System, Santa Cruz Biotechnology, Inc) per well to identify the P4E×6-induced SMAD phosphorylation signal after receptor binding of the P4 peptide. Whole cell protein homogenates (30 μg) were subjected to sodium dodecyl sulfate-polyacrylamide gel electrophoresis (SDS-PAGE) and transferred to Immobilon-P membranes (Millipore Co. Billerica, MA, USA). The membranes were incubated with various antibodies; anti-SMAD 1/5/9 antibody (1:500 dilution), Anti-SMAD1 + SMAD5 + SMAD9 (phospho S463 + S465 + S467) antibody [MMC-1-104-3] (1:1000 dilution) (Abcam), and anti-β-actin (Santa Cruz Biotechnology, Inc. CA, USA, 1:1000 dilution). The secondary antibodies are as follows; Horse Radish Peroxidase (HRP) conjugated Rabbit anti-Goat IgG for anti-SMAD and anti-pSMAD and anti-Mouse IgG Fc binding protein (m-IgG Fc BP) conjugated to HRP for anti-β actin.

4. *Fourier-transform infrared spectroscopy*

The prepared materials were analyzed using Fourier-transform infrared spectroscopy (FT-IR) imaging spectrometer system (Spectrum Two^{TM}, PerkinElmer).

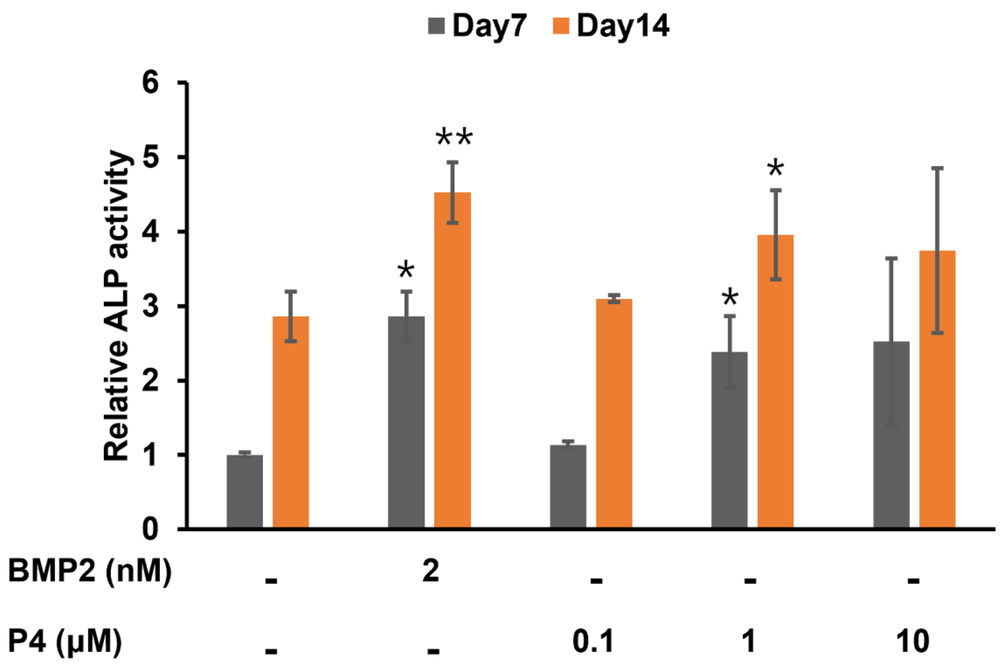

Supplementary Figure S1. Comparison of ALP activities of MC3T3 E1 cells treated with P4 peptide or BMP2 protein.

Comparison of ALP activity on Day 7 and on Day 14 after cell treatment for each indicated concentration of P4 and positive control BMP2 protein. ALP activity was expressed as a relative value to the activity on Day 7 of the control group to which nothing was added. The value is expressed as the mean ± SD. Statistically significant differences are indicated with * p <0.05 and ** p < 0.01 *vs* ALP activity of no addition control on Day 7 or Day 14.

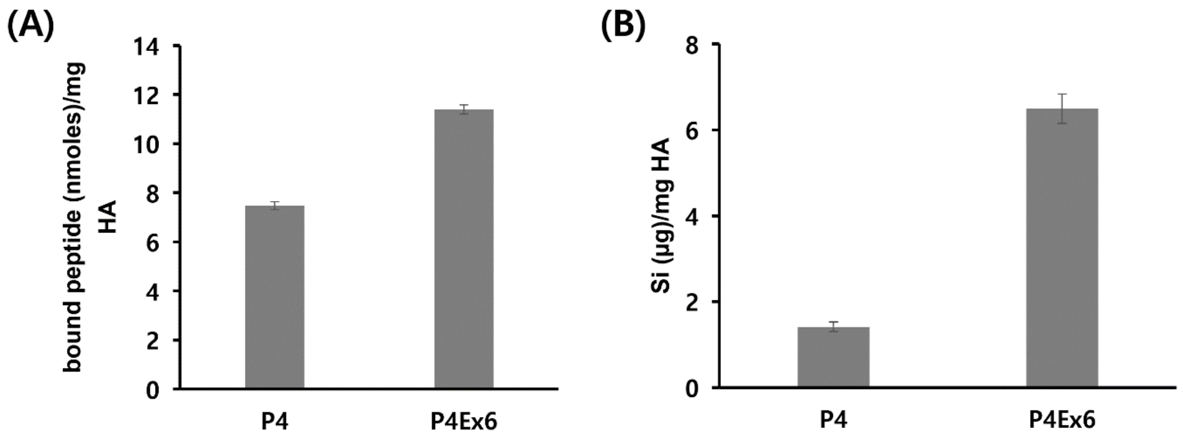

Supplementary Figure S2. Comparison of the bound amounts of peptide P4 and P4Ex6 for HA (A) and their ability to form silica after bonding (B).

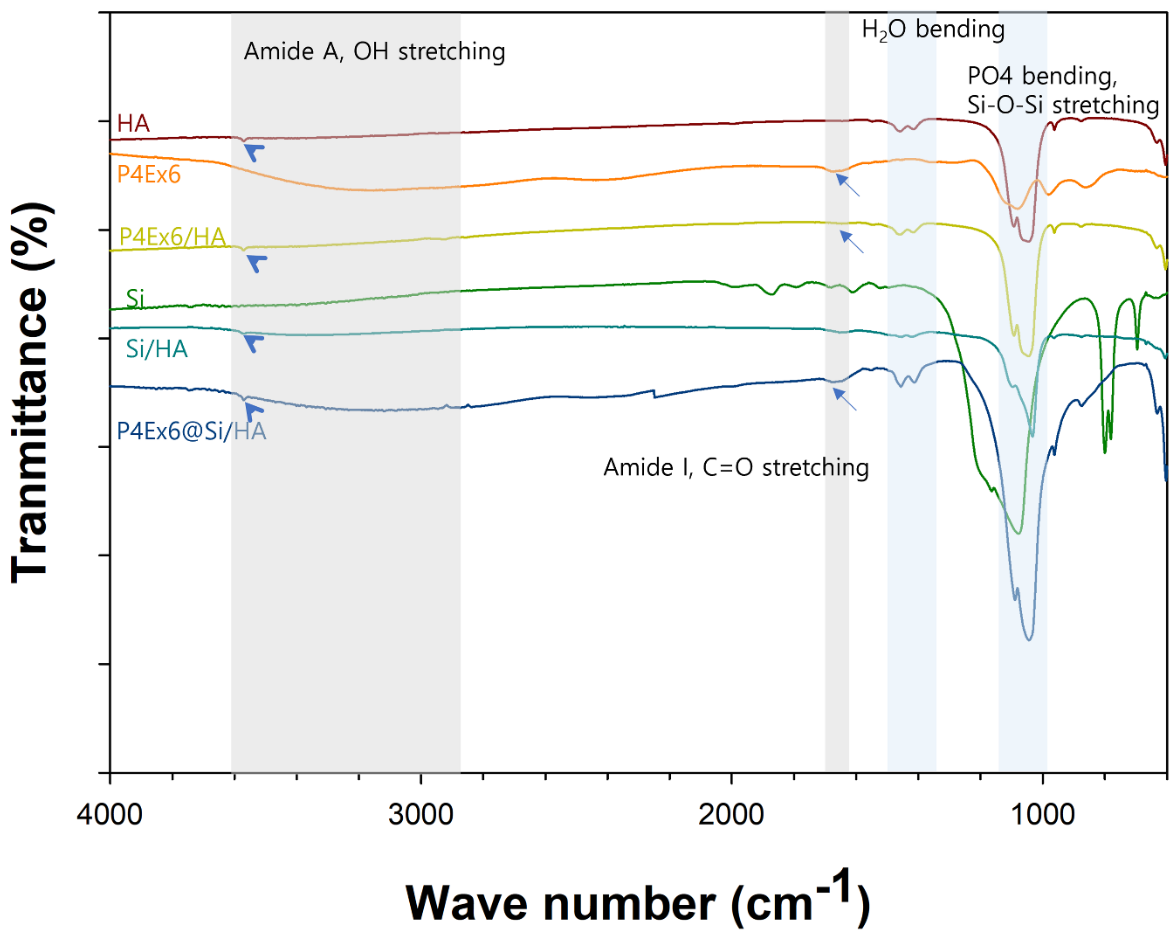

Supplementary Figure S3. Fourier transform infrared (FT-IR) spectra.

The FT-IR spectrum of hydroxyapatite revealed major absorption bands at approximately 604, 1045, and 1095 cm^{-1}, indicating PO_4 vibration. Additionally, the peak at 3574 cm^{-1} suggested OH stretching of HA, which appears all HA composites (arrow head, Fig.). The FT-IR spectrum of P4E×6 peptide displayed amide I at approximately 1675 cm^{-1} (C=O elongation) (arrow, Fig.). The FTIR data of peptide also indicated significant absorption bands at 1080 cm-1 (aliphatic amine, C-N stretching vibration) and at approximately 3000-3500 cm-1, which appears in all components, suggesting -OH vibration, and NH vibration. Symmetry and asymmetry stretching vibrations of Si-O-Si were observed at approximately 808cm-1 and 1077 cm-1, respectively. No specific peaks for each coating component were detected in the case of peptide-only and silica-only coating. However, in P4Ex6@Si/HA, amide I at 1651 cm^{-1} (arrow, Fig.) and -OH vibrations at around 3200-3500 cm-1 for the P4 peptide and silica, respectively were identified.

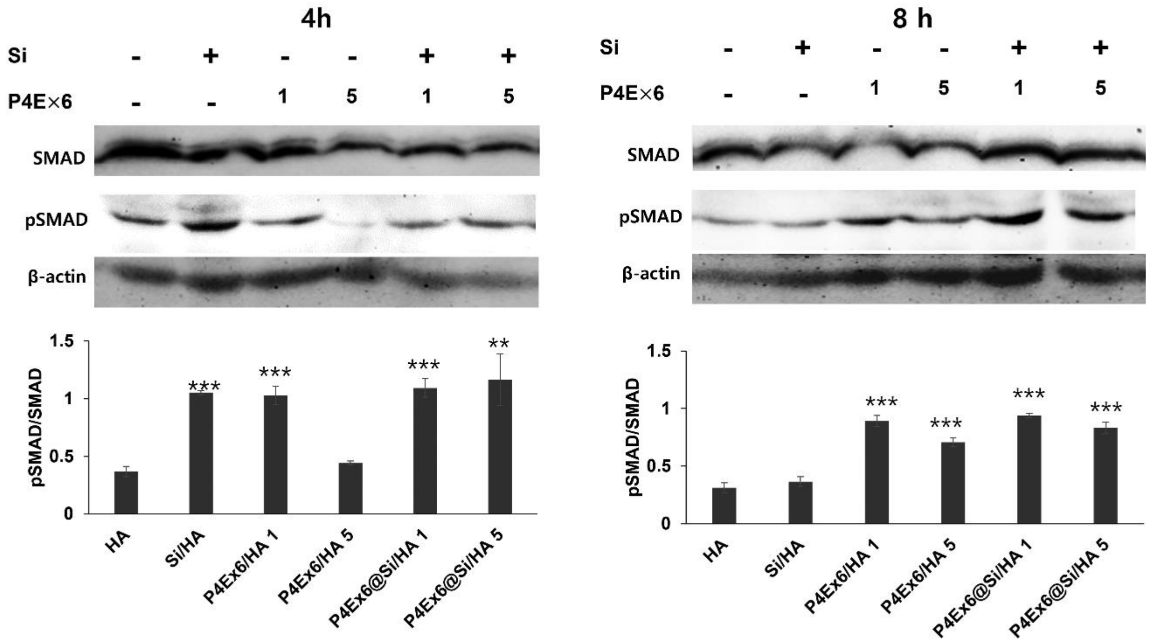

Supplementary Figure S4. SMAD signaling in MC3T3 E1 cells in response to indicated HA composites

MC3T3 E1 cells were exposed to each preparation without P4E×6 peptide or with 1µg or 5 µg of P4E×6 peptide for 4 h and 8 h, respectively. Quantitative expression of representative of SMAD 1/5/9 and its phosphorylated form in MC3T3 E1 cell whole lysate were determined by immunoblot analysis. Data are representative of two experiments. The expression of β–actin was used as loading control. The expression level of the quantitative phospho-SMAD is a relative value to the corresponding SMAD level. The value is expressed as the mean ± SD. Statistically significant differences are indicated with ** p <0.01 and *** p < 0.001 vs that of no addition control (HA) on 4 h or 8 h.
